# Supplementary material for: Spontaneous space closure after extraction of young first permanent molar. Retrospective cohort study
Source: PeerJ. 2024 Oct 23;12:e18276. doi: 10.7717/peerj.18276 (PMC11512553; doi:10.7717/peerj.18276)
Supplement: Supplemental Information 2 [file peerj-12-18276-s002.docx]

**Supplementary Table 1**. Dental characteristics of FPMs and SPMs prior to FPM extraction.

|  | | **Arch of extracted tooth** | | Total | P value |
| --- | --- | --- | --- | --- | --- |
| **Characteristics of FPMs** | | **Maxillary** | **Mandibular** |  |  |
| **SPM stage of development (Demirjian Stage)** | **Stage D** | 12 (36.4) | 21 (26.6) | 33 (29.5) | 0.400 |
|  | **Stage E** | 16 (48.5) | 38 (48.1) | 54 (48.2) |  |
|  | **Stage F/G** | 5 (15.2) | 20 (25.3) | 25 (22.3) |  |
| **Total** | | 33 (100.0) | 79 (100.0) | 112 (100.0 |  |
| **Coronal angulation of SPM before FPM extraction** | **Mesial** | 0 (0.0) | 71 (89.9) | 71 (63.4) | <0.001 |
|  | **Distal** | 28 (84.8) | 1 (1.3) | 29 (25.9)5 |  |
|  | **Perpendicular** | 5 (15.2) | 7 (8.9) | 12 (10.7) |  |
| **Total** | | 33 (100.0) | 79 (100.0) | 112 (100) |  |

FPM: First Permanent Molar, SPM, Second Permanent Molar

**Supplementary Table 2.** ABO combined clinical/radiographic grading parameters at the side of FPM extraction (N=112).

| **Combined ABO- parameters** | **Maxillary** | **Mandibular** | **Total** |
| --- | --- | --- | --- |
| **0** | 4 (12.1) | 1 (1.3) | 5 (4.5) |
| **1** | 10 (30.3) | 2 (2.5) | 12 (10.7) |
| **2** | 6 (18.2) | 13 (16.5) | 19 (17.0) |
| **3** | 5 (15.2) | 22 (27.8) | 27 (24.1) |
| **4** | 7 (21.2) | 21 (26.6) | 28 (25.0) |
| **5** | 1 (3.0) | 14 (17.7) | 15 (13.4) |
| **6** | 0 (0.0) | 6 (7.6) | 6 (5.4) |
| **Total** | 33 (100.0) | 79 (100.0) | 112 (100.0) |
