# Supplementary material for: Spontaneous space closure after extraction of young first permanent molar. Retrospective cohort study
Source: PeerJ. 2024 Oct 23;12:e18276. doi: 10.7717/peerj.18276 (PMC11512553; doi:10.7717/peerj.18276)
Supplement: Supplemental Information 3 [file peerj-12-18276-s003.docx]

| Hospital Centre | |
| --- | --- |
| 1 | UDH |
| 2 | KAMC |
| 3 | KFAFH |

| Gender | |
| --- | --- |
| 1 | Male |
| 2 | Female |

| Age at the time of treatment | |
| --- | --- |
| 1 | 7-8 years old |
| 2 | 9-10 years old |
| 3 | 11-13 years old |

| Arch of extracted tooth | |
| --- | --- |
| 1 | Maxillary |
| 2 | Mandibular |

| The Quadrant of The Extracted FPM | |
| --- | --- |
| 1 | 16 |
| 2 | 26 |
| 3 | 36 |
| 4 | 46 |

| Stage of Development of 2nd Molar (Demirjian Stage) Before Extraction | |
| --- | --- |
| 1 | Stage D |
| 2 | Stage E |
| 3 | Stage F |
| 4 | Stage G |

| The Angulation of 2nd Molar Before Extraction of FPM | |
| --- | --- |
| 1 | Mesial |
| 2 | Distal |
| 3 | Perpendicular |

| (Two Scores) Interproximal Contact Between 2nd Premolar and 2nd Molar in Extraction Side | |
| --- | --- |
| 0 | <0.5 mm |
| 1 | More than 0.5 mm |

| Severity of Interproximal Contact Between 2nd Premolar and 2nd Molar in mm in Extraction Side | |
| --- | --- |
| 0 | <0.5 mm |
| 1 | 0.5 to 1 mm |
| 2 | >1 mm |
| 3 | Not applicable |

| (Two Scores) Interproximal Contact Between Premolars in Extraction side | |
| --- | --- |
| 0 | <0.5 mm |
| 1 | More than 0.5 mm |

| Severity of Interproximal Contact Between Premolars in mm in Extraction Side | |
| --- | --- |
| 0 | <0.5 mm |
| 1 | 0.5 to 1 mm |
| 2 | >1 mm |
| 3 | Not applicable |

| Interproximal Contact Between 2nd Premolar and FPM in Non Extraction Side | |
| --- | --- |
| 0 | <0.5 mm |
| 1 | 0.5 to 1 mm |
| 2 | >1 mm |
| 3 | Not applicable |
| 4 | Contralateral FPM is extracted |

| Severity of Interproximal Contact Between 2nd Premolar and FPM in mm in Non Extraction Side | |
| --- | --- |
| 0 | <0.5 mm |
| 1 | 0.5 to 1 mm |
| 2 | >1 mm |
| 3 | Not applicable |
| 4 | Contralateral FPM is extracted |

| Interproximal Contact Between Premolars in Non Extraction Side | |
| --- | --- |
| 0 | <0.5 mm |
| 1 | 0.5 to 1 mm |
| 2 | >1 mm |
| 3 | Not applicable |
| 4 | Contralateral FPM is extracted |

| Severity of IPC Between Premolars in mm in Non Extraction Side | |
| --- | --- |
| 0 | <0.5 mm |
| 1 | 0.5 to 1 mm |
| 2 | >1 mm |
| 3 | Not applicable |
| 4 | Contralateral FPM is extracted |

| (Two Scores) Alignment/Rotation of 2nd Premolar In Extraction Side | |
| --- | --- |
| 0 | <0.5 mm |
| 1 | more than 0.5 mm |

| Severity of Alignment/Rotation Of 2nd Premolar in mm in Extraction Side | |
| --- | --- |
| 0 | <0.5 mm |
| 1 | 0.5 to 1 mm |
| 2 | >1 mm |
| 3 | Not applicable |

| (Two Scores) Alignment/Rotation of 2nd Molar In Extraction Side | |
| --- | --- |
| 0 | <0.5 mm |
| 1 | more than 0.5 mm |

| Severity of Alignment of 2nd Molar in mm In Extraction Side | |
| --- | --- |
| 0 | <0.5 mm |
| 1 | 0.5 to 1 mm |
| 2 | >1 mm |
| 3 | Not applicable |

| Alignment/Rotation of 2nd Premolar In Non Extraction Side | |
| --- | --- |
| 0 | Off <0.5 mm |
| 1 | Off 0.5 to 1 mm |
| 2 | Off>1 mm |
| 3 | Not applicable |
| 4 | Contralateral FPM is extracted |

| Severity of Alignment/Rotation of 2nd Premolar in mm In Non Extraction Side | |
| --- | --- |
| 0 | <0.5 mm |
| 1 | 0.5 to 1 mm |
| 2 | >1 mm |
| 3 | Not applicable |
| 4 | Contralateral FPM is extracted |

| Alignment/Rotation of FPM In Non Extraction Side | |
| --- | --- |
| 0 | Off <0.5 mm |
| 1 | Off 0.5 to 1 mm |
| 2 | Off>1 mm |
| 3 | Not applicable |
| 4 | Contralateral FPM is extracted |

| Severity of Alignment/Rotation of FPM in mm In Non Extraction | |
| --- | --- |
| 0 | <0.5 mm |
| 1 | 0.5 to 1 mm |
| 2 | >1 mm |
| 3 | Not applicable |
| 4 | Contralateral FPM is extracted |

| (Two Scores) Marginal Ridge Between 2nd Premolar and 2nd Molar In Extraction Side | |
| --- | --- |
| 0 | <0.5 mm |
| 1 | more than 0.5 mm |

| Severity of Marginal Ridge Between 2nd Premolar and 2nd Molar in mm In Extraction Side | |
| --- | --- |
| 0 | <0.5 mm |
| 1 | 0.5 to 1 mm |
| 2 | >1 mm |
| 3 | Not applicable |

| Marginal Ridge Between 2nd Premolar and FPM In Non Extraction Side | |
| --- | --- |
| 0 | <0.5 mm |
| 1 | 0.5 to 1 mm |
| 2 | >1 mm |
| 3 | Not applicable |
| 4 | Contralateral FPM is extracted |

| Severity of Marginal Ridge Between 2nd Premolar and FPM in mm In Non Extraction Side | |
| --- | --- |
| 0 | <0.5 mm |
| 1 | 0.5 to 1 mm |
| 2 | >1 mm |
| 3 | Not applicable |
| 4 | Contralateral FPM is extracted |

| (Two Scores)Buccolingual Inclination of 2nd Molar In Extraction Side | |
| --- | --- |
| 0 | 0 to 1 mm |
| 1 | More than 1 mm |

| Severity of Buccolingual Inclination of 2nd Molar in mm In Extraction Side | |
| --- | --- |
| 0 | 0 to 1 mm |
| 1 | 1 to 2 mm |
| 2 | >2 mm |
| 3 | Not applicable |

| Buccolingual Inclination of FPM In Non Extraction Side | |
| --- | --- |
| 0 | Off 0 to 1 mm |
| 1 | Off 1 to 2 mm |
| 2 | Off > 2 mm |
| 3 | Not applicable |
| 4 | Contralateral FPM is extracted |

| Severity of Buccolingual Inclination of FPM in mm in Non Extraction Side | |
| --- | --- |
| 0 | 0 to 1 mm |
| 1 | 1 to 2 mm |
| 2 | > 2 mm |
| 3 | Not applicable |
| 4 | Contralateral FPM is extracted |

| (Two Scores)Root Angulation of 2nd Molar In Extraction Side | |
| --- | --- |
| 0 | Root Parallel |
| 1 | Root Not Parallel |

| Side of angulation of 2nd Molar In Extraction | |
| --- | --- |
| 0 | Perpendicular |
| 1 | Mesial |
| 2 | Distal |

| Root Angulation of FPM In Non Extraction Side | |
| --- | --- |
| 0 | Root Parallel |
| 1 | Root Not Parallel |
| 2 | Root contacting adjacent root |
| 3 | Not applicable |
| 4 | Contralateral FPM is extracted |

| Side of Angulation of FPM In Non Extraction Side | |
| --- | --- |
| 0 | Perpendicular |
| 1 | Mesial |
| 2 | Distal |
| 4 | Contralateral FPM is extracted |

| combined score grouped to favour completely perfect alignment _not | |
| --- | --- |
| 0 | Completely favour alignment |
| 1 | Completely unfavour alignment |

| Demirigian in two groups | |
| --- | --- |
| 1 | D and E |
| 2 | F and G |

| Demirigian in three groups | |
| --- | --- |
| 1 | D |
| 2 | E |
| 3 | F&G |
